# A Multiomics Assessment of Preoperative Exercise in Pancreatic Cancer Survivors Receiving Neoadjuvant Therapy: A Case Series

**DOI:** 10.3390/pathophysiology31010013

**Published:** 2024-03-20

**Authors:** Travis Nemkov, Francesca Cendali, Monika Dzieciatkowska, Daniel Stephenson, Kirk C. Hansen, Catherine M. Jankowski, Angelo D’Alessandro, Ryan J. Marker

**Affiliations:** 1 Department of Biochemistry and Molecular Genetics, Anschutz Medical Campus, University of Colorado, Aurora, CO 80045, USA; francesca.cendali@cuanschutz.edu (F.C.); monika.dzieciatkowska@cuanschutz.edu (M.D.); daniel.stephenson@cuanschutz.edu (D.S.); angelo.dalessandro@cuanschutz.edu (A.D.); 2 College of Nursing, University of Colorado, Aurora, CO 80045, USA; catherine.jankowski@cuanschutz.edu; 3 Department of Medicine, Anschutz Medical Campus, University of Colorado, Aurora, CO 80045, USA

**Keywords:** pancreatic cancer, exercise oncology, pancreatic adenocarcinoma, metabolomics, lipidomics, proteomics, biomarkers

## Abstract

To molecularly characterize the impact of exercise on mitigating neoadjuvant treatment (NAT)-induced physical decline in pancreatic ductal adenocarcinoma (PDAC) patients, a multi-omics approach was employed for the analysis of plasma samples before and after a personalized exercise intervention. Consisting of personalized aerobic and resistance exercises, this intervention was associated with significant molecular changes that correlated with improvements in lean mass, appendicular skeletal muscle index (ASMI), and performance in the 400-m walk test (MWT) and sit-to-stand test. These alterations indicated exercise-induced modulation of inflammation and mitochondrial function markers. This case study provides proof-of-principal application for multiomics-based assessments of supervised exercise, thereby supporting this intervention as a feasible and beneficial intervention for PDAC patients to potentially enhance treatment response and patient quality of life. The molecular changes observed here underscore the importance of physical activity in cancer treatment protocols, advocating for the development of accessible multiomics-guided exercise programs for cancer patients.

## 1. Introduction

Pancreatic cancer is the fourth leading cause of cancer-related death in the United States [1] and surgical resection is the best-known treatment [2]. Neoadjuvant treatment (NAT) is often prescribed to downstage tumors and improve response to surgical resection [3]. This preoperative treatment, however, can decrease patient physical fitness [4] and muscle mass [5]. As both of these measures are positively associated with improved surgical outcomes and survival, interventions designed to reduce these negative responses should be developed to improve surgical outcomes [5,6]. Supervised exercise has been shown to improve cardiovascular fitness and muscle mass in survivors (in accordance with the National Cancer Institute definition referring to an individual from the time of cancer diagnosis through the rest of life) of other cancer types receiving NAT [7,8,9], directly counteracting the negative effects of the treatment. Unfortunately, due to logistical limitations such as travel restrictions and socioeconomic barriers preventing access to specialized facilities offering supervised exercise programs, only a few studies have been conducted on supervised exercise during NAT in patients with pancreatic cancer [10,11,12].

A recent systematic review of investigations into preoperative fitness in patients with pancreatic cancer identified only six trials published to date [13]. Four of these trials were in patients receiving NAT and only one provided supervised exercise, performed by several of the current authors [14], with others providing lower-intensity unsupervised alternatives. The primary finding of this review was that exercise in pancreatic cancer survivors receiving NAT was safe and feasible, with no significant adverse events reported in any of the investigations. Improvements in muscle mass [14], fitness [12], and surgical outcomes [15] were found in some studies but were not consistent across studies. While the study of supervised exercise was limited to three participants, several meaningful changes in fitness and muscle mass were observed. Of particular note were results related to pancreatic cancer cachexia. Two of the three participants met objective definitions of pancreatic cancer cachexia (determined by dual-energy X-ray absorptiometry [DXA]) [16] at baseline, but not after the exercise intervention, despite receiving NAT. This result bears further investigation given its potential clinical implications. In particular, recent advances in high-throughput mass spectrometry [17] allow for plasma-based multi-omics investigation of the physiological mechanisms promoting increased muscle mass and cardiometabolic fitness, providing insight to inform future exercise interventions in clinical populations.

Plasma-based metabolomics, lipidomics, and proteomics have been performed in elite and recreational athletes to characterize molecular responses to exercise [18,19,20,21,22,23]. Targeted analyses can reveal adaptations to exercise using markers of mitochondrial function (pyruvate, lactate, citrate, succinate, and malate), fatty acid metabolism (free- and carnitine-conjugated mono-, poly-, and highly unsaturated fatty acids), muscle mass, and performance (myoglobin, creatine kinase M, leptin, C-C motif chemokine 28, and metalloproteinase inhibitor 4) [24]. As metabolic reprogramming and mitochondrial dysfunction are central hallmarks of cancer [25], the rationale behind exercise as a therapeutic intervention in this population lies in the opportunity to boost metabolic health and mitochondrial biogenesis, which collectively function as a co-adjuvant to more established iatrogenic interventions. While some of these methods have recently been implemented in other cancer populations receiving NAT [26], there are currently no investigations in survivors with pancreatic cancer, or in conjunction with an exercise intervention. This investigation provides proof-of-concept and preliminary results from multi-omics analyses of banked plasma samples from participants in a previously reported case series of a supervised exercise program during NAT in patients with pancreatic cancer [14].

## 2. Materials and Methods

### 2.1. Participants, Assessments, and Intervention

This trial has been previously reported, including in-depth descriptions of patients, assessments, exercise intervention, and outcomes (body composition, physical function, patient-reported outcomes) [14]. All participants were functionally able to complete all fitness and functional assessments along with a personalized exercise intervention at intake. All were recently diagnosed (<4 weeks) with non-metastatic borderline-resectable pancreatic adenocarcinoma upon enrollment, prescribed NAT, and living in the vicinity of the exercise facility on the University of Colorado Anschutz Medical Campus. Participants 1 and 3 reported regular exercise prior to diagnosis and Participant 2 reported regular but declining physical activity over the past several years. All participants received standard-of-care interventions in addition to the exercise intervention, which included appointments with a registered dietician. Participants 1 and 2 eventually underwent open Whipple procedures. Prior to surgery, it was discovered that Participant 3′s cancer had metastasized, and she was transitioned to palliative care.

The exercise intervention was performed during NAT. Participants received 2–3 supervised 60 min personalized exercise sessions per week until surgery, working one-on-one with an exercise physiologist. Sessions consisted of a 10 min cardiovascular warmup, followed by 45 min of combined aerobic and resistance exercises, and ended with 5 min of flexibility activities. Exercise intensity was determined by a 0–10 rating of perceived exertion (RPE) scale, with a target of 7/10. Heart rate was monitored (Polar F4, Kempele, Finland) and kept below 85% heart rate reserve. Prior to each exercise session, participants provided information on changes in symptoms or the onset of new symptoms, which guided exercise modification to facilitate maximum symptom-limited participation. Assessments were performed at baseline, pre-surgery, and six weeks post-surgery. No intervention was delivered after surgery. Assessments included multiple measures of body composition (via DXA), physical fitness and function, and patient-reported outcomes.

### 2.2. Plasma Samples

Blood samples were collected in EDTA tubes at each timepoint during either clinical blood draws within one week of the assessment or a study visit on the day of the assessment (the baseline sample was collected in close proximity to multidisciplinary reviews). Plasma was separated and stored at −80 °C for approximately four years without thaw.

### 2.3. Metabolomics and Lipidomics Sample Preparation

Prior to metabolomics or lipidomics analysis, individual samples were placed in 1.5 mL tubes for either metabolite or lipid extraction. Twenty µL were suspended in 180 µL of water/methanol (50:50 *v*/*v*) for metabolite extraction or 180 µL of isopropanol/methanol (50:50 *v*/*v*) for lipid extraction. Suspensions were vortexed for 30 min at 4 °C and then centrifuged for 10 min, 18,213× *g*, 4 °C. Supernatants were isolated for LC-MS.

### 2.4. Metabolomics and Lipidomics UHPLC-MS Data Acquisition and Processing

Analyses were performed as previously published [27]. Briefly, the analytical platform employs a Vanquish UHPLC system (Thermo Fisher Scientific, San Jose, CA, USA) coupled online to a Q Exactive mass spectrometer (Thermo Fisher Scientific, San Jose, CA, USA). Polar metabolite extracts were resolved in singlicate over a Kinetex C18 column, 2.1 mm × 150 mm, 1.7 µm particle size (Phenomenex, Torrance, CA, USA) equipped with a guard column (SecurityGuard^TM^ Ultracartridge—UHPLC C18 for 2.1 mm ID Columns, Phenomenex, Torrance, CA, USA) using an aqueous phase (A) of water and 0.1% formic acid and a mobile phase (B) of acetonitrile and 0.1% formic acid for positive ion polarity mode, and an aqueous phase (A) of water:acetonitrile (95:5) with 1 mM ammonium acetate and a mobile phase (B) of acetonitrile:water (95:5) with 1 mM ammonium acetate for negative ion polarity mode. The column was equilibrated at 5% B, and upon injection of 10 μL of extract, samples were eluted from the column using the solvent gradient: 0.5–1.1 min 5–95% B at 0.45 mL/min; hold at 95% B for 1.65 min at 0.45 mL/min, and then decrease to 5% over 0.25 min at 0.45 mL/min, followed by a re-equilibration hold at 5% B for 2 min at 0.45 mL/min. The Q Exactive mass spectrometer (Thermo Fisher Scientific, San Jose, CA, USA) was operated independently in positive or negative ion mode, scanning in Full MS mode (2 μscans) from 60 to 900 *m*/*z* at 70,000 resolution, with 4 kV spray voltage, 45 sheath gas, 15 auxiliary gas, AGC target = 3e6, maximum IT = 200 ms. Non-polar lipid extracts were resolved over an ACQUITY HSS T3 column, 2.1 mm × 150 mm, 1.8 µm particle size (Waters, MA, USA) using an aqueous phase (A) of 25% acetonitrile and 5 mM ammonium acetate and a mobile phase (B) of 90% isopropanol, 10% acetonitrile, and 5 mM ammonium acetate. The column was equilibrated at 30% B, and upon injection of 10 μL of extract, samples were eluted from the column using the solvent gradient: 0–9 min 30–100% B at 0.325 mL/min; hold at 100% B for 3 min at 0.3 mL/min, and then decrease to 30% over 0.5 min at 0.4 mL/min, followed by a re-equilibration hold at 30% B for 2.5 min at 0.4 mL/min. The Q Exactive mass spectrometer was operated in positive ion mode, scanning in Full MS mode (2 μscans) from 150 to 1500 *m*/*z* at 70,000 resolution, with 4 kV spray voltage, 45 sheath gas, 15 auxiliary gas. When required, dd-MS2 was performed at 17,500 resolution, AGC target = 1e5, maximum IT = 50 ms, and stepped NCE of 25, 35 for positive mode, and 20, 24, and 28 for negative mode. Calibration was performed prior to analysis using the Pierce^TM^ Positive and Negative Ion Calibration Solutions (Thermo Fisher Scientific).

### 2.5. Metabolomics and Lipidomics Data Analysis

Acquired data were converted from raw to mzXML file format using RawConverter [28]. Samples were analyzed in randomized order with a technical mixture injected after every 10 samples to qualify instrument performance. Metabolite assignments were performed using accurate intact mass (sub-10 ppm), isotopologue distributions, and retention time/spectral comparison to an in-house standard compound library (MSMLS, IROA Technologies, Ann Arbor, MI, USA) using El-MAVEN (Elucidata, Cambridge, MA, USA). Lipidomics data were analyzed using LipidSearch 5.0 (Thermo Fisher Scientific, San Jose, CA, USA), which provides lipid identification on the basis of accurate intact mass, isotopic pattern, and fragmentation pattern to determine lipid class and acyl chain composition. Graphs, heat maps and statistical analyses (either *t*-test or ANOVA), multivariate analyses including Principal Component Analysis (PCA), Partial Least Squares-Discriminant Analysis (PLS-DA), hierarchical clustering analysis (HCA), and metabolite pathway enrichment analysis were performed using MetaboAnalyst 5.0 [29].

### 2.6. Inductively-Coupled Plasma (ICP) Mass Spectrometry

Samples were analyzed as previously published [30]. Prior to ICP-MS analysis, 10 μL of plasma was aliquoted into a 15 mL conical tube. A total of 200 μL of 65% nitric acid and 20 ng/mL of gold were added to each sample followed by an addition of 100 μL of 30% hydrogen peroxide and brief vortexing. Samples were then incubated in an oven at 70 °C for approximately 2 h. Following incubation, 2190 μL of MilliQ water was added to each tube (final nitric acid percentage of ~5%) and all samples were vortexed briefly. All samples were then diluted 1:15 in a solution consisting of 20 ng/mL and 5% nitric acid. Final dilutions of 1:250 and 1:3750 were then analyzed via ICP-MS. Different dilutions were used to ensure all analytes fell within the calibration curves. All chemicals and materials used for ICP-MS analysis were obtained from Thermo Fisher and all ICP-MS calibrants and solutions were obtained from SPEX CertiPrep (Cole Parmer, Vernon Hills, IL, USA). All samples were analyzed on a Thermo Scientific iCAP RQ ICP-MS coupled to an ESI SC-4DX FAST autosampler system utilizing a peristaltic pump. The optimization of the system was performed before the run by first calibrating the system with ICP-MS iCAP Q/Qnova Calibration Solution, Specpure (Thermo Fisher Scientific, San Jose, CA, USA). The system was subsequently tuned using a tuning solution consisting of Ba, Bi, Ce, Co, In, Li, and U at 1.00 ± 0.05 μg/L. To monitor performance while the system was running, we continually pumped the internal standard mix via the peristaltic pump and monitored the signal throughout the run. Additionally, quality controls of a known concentration of each analyte were injected at the beginning, throughout the run between samples, and at the end of the run. The acceptance criteria for all QCs were ±25% of the known concentration. Thermo Scientific Qtegra 2.10 software was used for all data acquisition and analysis.

### 2.7. Proteomics Sample Preparation

Individual samples were aliquoted (10 µL) 1.5 mL tube and resuspended in 180 µL of 5% SDS, sonicated for 1 h, and centrifuged for 5 min at 10,000× *g* to collapse foam. Proteins were digested in an S-Trap filter (Protifi, Huntington, NY, USA), following the manufacturer’s procedure. Briefly, samples were reduced with 10 mM dithiothreitol at 55 °C for 30 min, cooled to room temperature, and then alkylated with 25 mM iodoacetamide in the dark for 30 min. Afterward, phosphoric acid was added to the samples to a final concentration of 1.2% followed by 6 volumes of binding buffer (90% methanol; 100 mM triethylammonium bicarbonate (TEAB); pH 7.1). After gentle mixing, the protein solution was loaded onto an S-Trap filter, spun at 2000× *g* for 1 min, and the flow-through collected and reloaded onto the filter. This step was repeated three times, and then the filter was washed with 200 μL of binding buffer three times. Finally, 1 μg of sequencing-grade trypsin and 150 μL of digestion buffer (50 mM TEAB) were added onto the filter and digested at 47 °C for 1 h. To elute peptides, three stepwise buffers are applied, with 200 μL of each with one more repeat; these included 50 mM TEAB, 0.2% formic acid in water, and 50% acetonitrile and 0.2% formic acid in water. The peptide solutions were pooled, lyophilized, and resuspended in 0.1% formic acid.

### 2.8. Proteomics Data Acquisition and Processing

A total of 200 ng of each sample was loaded onto individual Evotips (Evosep, Odense, Denmark) for desalting and then washed with 20 μL 0.1% formic acid followed by the addition of 100 μL of storage solvent (0.1% formic acid) to keep the Evotips wet until analysis. The Evosep One system (Evosep, Odense, Denmark) was coupled to the timsTOF Pro mass spectrometer (Bruker Daltonics, Bremen, Germany). Data were collected over an *m*/*z* range of 100 to 1700 for MS and MS/MS on the timsTOF Pro instrument using an accumulation and ramp time of 100 ms. Post-processing was performed with PEAKS studio (Version X+, Bioinformatics Solutions Inc., Waterloo, ON, Canada). Pathway analyses were performed with the DAVID 2021 software v2023q4. Graphs and statistical analyses were prepared with GraphPad Prism 8.0 (GraphPad Software, Inc., La Jolla, CA, USA), and MetaboAnalyst 5.0 [29].

### 2.9. Feature Enrichment Analyses

Features that had a magnitude Pearson Correlation R > 0.7 with target metrics (i.e., lean mass, appendicular skeletal muscle index (ASMI), 400 m walk test distance, and 30 s sit-to-stand test repetitions) were included for enrichment analyses. Metabolites were analyzed by the Pathway Analysis module of MetaboAnalyst 5.0, using SMPDB for enrichment analysis. Proteins were analyzed by GO Enrichment Analysis using PANTHER [31]. Enrichment plots were generated with ggplot v3.5.0. Lipid enrichment analyses were performed using BioPAN [32].

## 3. Results

**Figure 1:**
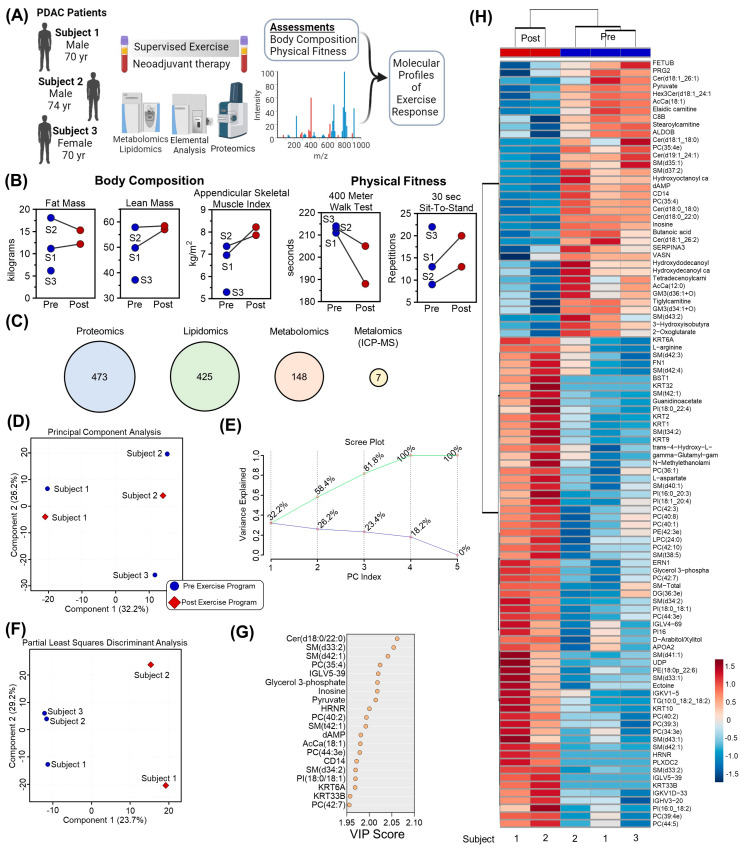
Multiomics analysis of plasma before and after a 12–week supervised exercise program for patients with pancreatic adenocarcinoma tumors. (**A**) Three participants were prescribed a 12–week supervised exercise program. Plasma samples were collected from all three participants before training, and two of the participants upon completion of training. (**B**) Body composition and physical fitness parameters are shown for individual participants (labeled P and corresponding number) in blue or red circles for Pre and Post exercise program, respectively, along with (**C**) a multiomics platform consisting of proteomics, lipidomics, metabolomics, and metalomics. The number of analytes included in the study is numerated in the respective circles. (**D**) Principal Component Analysis of multiomics data in blue circles or red diamonds for Pre or Post, respectively, along with (**E**) the corresponding Scree plot. (**F**) Partial Least Squares Discriminant Analysis (PLS–DA) along with (**G**) the top 15 features containing the largest variable importance in projection (VIP) scores of the PLS–DA. (**H**) The top 100 significant features are plotted as a heat map and colored according to Z–score.

Three participants were enrolled in this study, with body composition measurements provided in Table 1. Changes in multiple outcomes measures during the intervention were observed, including cardiovascular fitness: 400 m walk test and 30 s sit-to-stand test, and body composition: total mass, lean mass, fat mass, appendicular lean mass, appendicular skeletal muscle index [ASMI] (Table 2). Plasma was collected from three participants at baseline and two participants after completing a personalized supervised exercise program (Figure 1A). In addition to physiological and functional assessments [14] including lean mass, appendicular skeletal muscle index (ASMI), 400 m walk test distance, and 30 s sit-to-stand test results (Figure 1B), samples were analyzed using mass spectrometry-based proteomics, lipidomics, metabolomics, and metalomics (using ICP-MS). After raw data processing and filtering, 473, 425, 148, and 7 features were included in subsequent statistical analyses, respectively (Figure 1C, Supplementary Table S1). Unsupervised Principal Component Analysis of the consolidated dataset separated the three participants into distinct regions of the plot, highlighting that chemical individuality drives the majority of distinction between samples (Figure 1D and accompanying Scree Plot in Figure 1E). Incorporation of partial supervision for the development of a Partial Least Squares Discriminant Analysis model to describe differences in plasma before and after the 12-week exercise program separated samples based on timepoint along Component 1, explaining 23.7% of the sample variance (Figure 1F). The top contributors to this clustering pattern by Variable Importance in Project (VIP) included lipids (ceramides, Cer; sphingomyelins, SM; phosphocholines, PC; and acylcarnitines, AcCa), metabolites (pyruvate, inosine, glycerol 3-phosphate, dAMP), and proteins (IGLV5-39, HRNR, CD14) (Figure 1G). Collectively, the relative levels of the top 100 significant features by T-test were able to cluster the Pre and Post samples by Hierarchical Clustering Analysis (Figure 1H).

**Figure 2:**
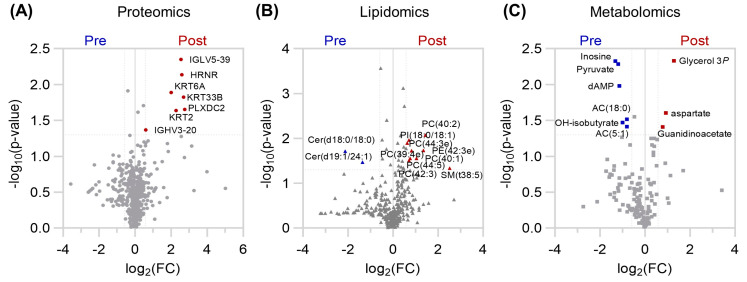
Volcano Plots of (**A**) proteomics (circles), (**B**) lipidomics (triangles), and (**C**) metabolomics (squares) results for Participants 1 and 2 Pre and Post exercise intervention in blue and red, respectively. The x–axis indicates log2(fold change[FC]) and the y–axis -log10(*p*–value) from a two-tailed paired T–test. Features with FC > 1 and *p*-value < 0.05 are colored according to positive (red) or negative (blue) fold change.

Analysis of the protein, lipid, and metabolite compartments individually also revealed subtle, but significantly changing levels (Figure 2). Only the levels of immunoglobulin fragments (IGLV5-39, IGHV3-20), hornerin (HRNR), keratin (KRT2, KRT6A, KRT33B), plexin domain containing 2 (PLXDC2) were higher after the exercise program (Figure 2A). Increases in phosphatidylinositol (PI), phosphoethanolamine (PE), PC, and SM lipid species were observed, along with decreased levels of two ceramides (Figure 2B). Furthermore, the lipid backbone glycerol 3-phosphate, along with aspartate and the creatine precursor guanidinoacetate were elevated in plasma, while the nucleoside inosine, glycolytic end product pyruvate, ketone body hydroxybutyrate, and branched chain/fatty acid oxidation intermediates acylcarnitines AC(5:1) and AC(18:0), were diminished after training (Figure 2C).

**Figure 3:**
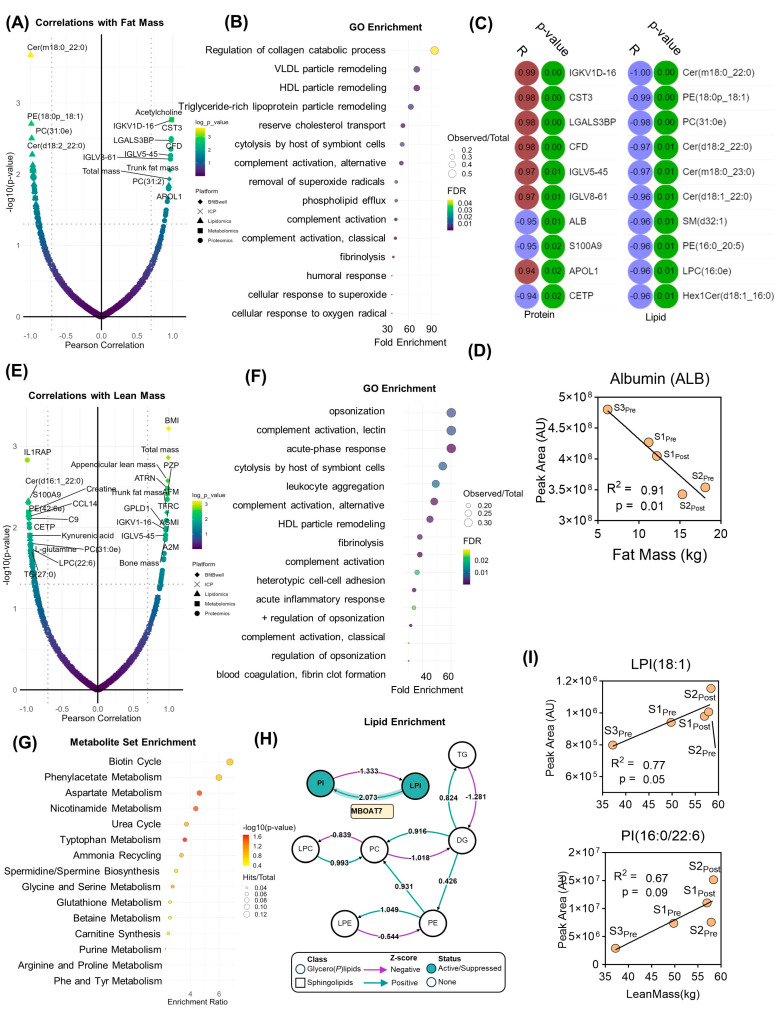
Correlation analysis with fat and lean mass. (**A**) Total feature correlations with fat mass. (**B**) Gene ontology (GO) enrichment of proteomics data and (**C**) Pearson correlation coefficients and *p*–values for the top 10 protein (left) and lipid (right) correlates. (**D**) Correlation plots are shown for cytastatin 3, albumin, and the total ceramide pool. (**E**) Correlations with lean mass are shown as well as (**F**) enriched GO, (**G**) metabolite, and (**H**) lipid pathways. (**I**) Correlation plots for lysophosphatidylinosital(18:1) and PI(16:0/22:6).

In addition to the measurement of steady-state protein, lipid, and metabolite levels, systems analyses were performed to identify molecular clusters that correlate with physiological and performance parameters measured before and after the exercise program. Molecular features that significantly correlated (Pearson |R| > 0.7, log_10_(*p*-value) > 1.3) with fat mass were identified (Figure 3A). This list was then filtered by proteins for subsequent gene ontology (GO) (Figure 3B), highlighting an enrichment in very low-density lipoprotein (VLDL) and high-density lipoprotein (HDL) particle remodeling, lipid transport, an immune system function (Figure 3B). While only two metabolites were statistically correlated with fat mass change (leucine, acetylcholine, Supplementary Table S1), 22 proteins and 33 lipids did significantly correlate (top 10 for each shown in Figure 3C). While cytastatin 3 was a top protein correlate, albumin was the top negative correlate (Figure 3D). Lipid levels, especially ceramides predominantly correlated inversely with fat mass (Figure 3D). On the other hand, correlates with lean mass (Figure 3E) and GO enrichment revealed that proteins correlating with changes in lean mass were functionally important in immune system processes, including the complement pathway, acute phase response, and leukocyte activation (Figure 3F). Metabolite set enrichment (MSEA) highlighted metabolites associated with amino acid and nitrogen metabolism (urea cycle, ammonium recycling, polyamine biosynthesis) (Figure 3G). Lipid enrichment analysis demonstrated an active network centered upon (lyso)phosphatidylinositol (LPI, PI) homeostasis mediated in part by the lysophospholipid acyltransferase, MBOAT7 (Figure 3H). In support of enhanced MBOAT7 activity, both LPI(18:1) and PI(16:0/22:6) were positively associated with increased lean mass (Figure 3I).

**Figure 4:**
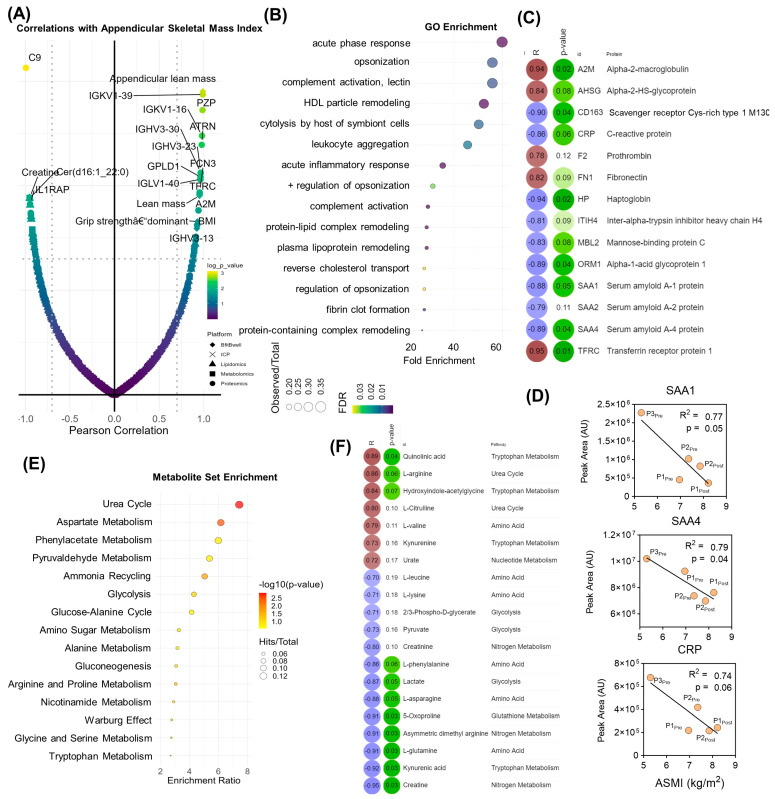
Correlation analysis with Appendicular Skeletal Mass Index. (**A**) Total feature correlations with appendicular skeletal mass index. (**B**) Gene ontology (GO) enrichment of proteomics data and (**C**) top protein Pearson correlation coefficients (positive correlations in red, negative correlations in blue) and associated *p*–values (indicated in green). (**D**) Correlations between ASMI and serum amyloid proteins 1 SAA1) and 4 (SAA4), and C Reactive Protein (CRP). (**E**) Metabolite set enrichment of metabolomics data, (**F**) top metabolite Pearson correlation coefficients and associated *p*-values.

These findings were mirrored when analyzing molecular correlates with appendicular skeletal muscle index (ASMI), which is derived from the lean mass measurements. Top correlates (Figure 4A) and GO enrichment (Figure 4B,C) highlighted the centrality of inflammation and the acute phase response, along with nitrogen metabolism. Individual correlation plots are shown for top inflammatory protein correlates serum amyloid A1 (SAA1) and 4 (SAA4) as well as C-reactive protein (CRP) (Figure 4D). MSEA revealed enrichment in the urea cycle and associated metabolites including arginine, citrulline, creatinine, creatine, asymmetric dimethyl arginine, and creatine (Figure 4D,E). Indeed, the levels of acute phase response proteins including alpha-2-macroglobulin, prothrombin, fibronectin, and transferrin receptor protein 1 were positively associated with increases in ASMI, while proinflammatory markers such as C-reactive protein, haptoglobin, and serum amyloid A1 and A4 were inversely associated (Figure 4C). Additionally, baseline circulating levels of arginine and citrulline, the respective substrate and product of nitric oxide synthase (NOS) were positively correlated along with the quinolinic/kynurenic acid ratio. Creatine, amino acids (glutamine, phenylalanine, asparagine), and lactate were inversely correlated with an increase in ASMI (Figure 3E).

**Figure 5:**
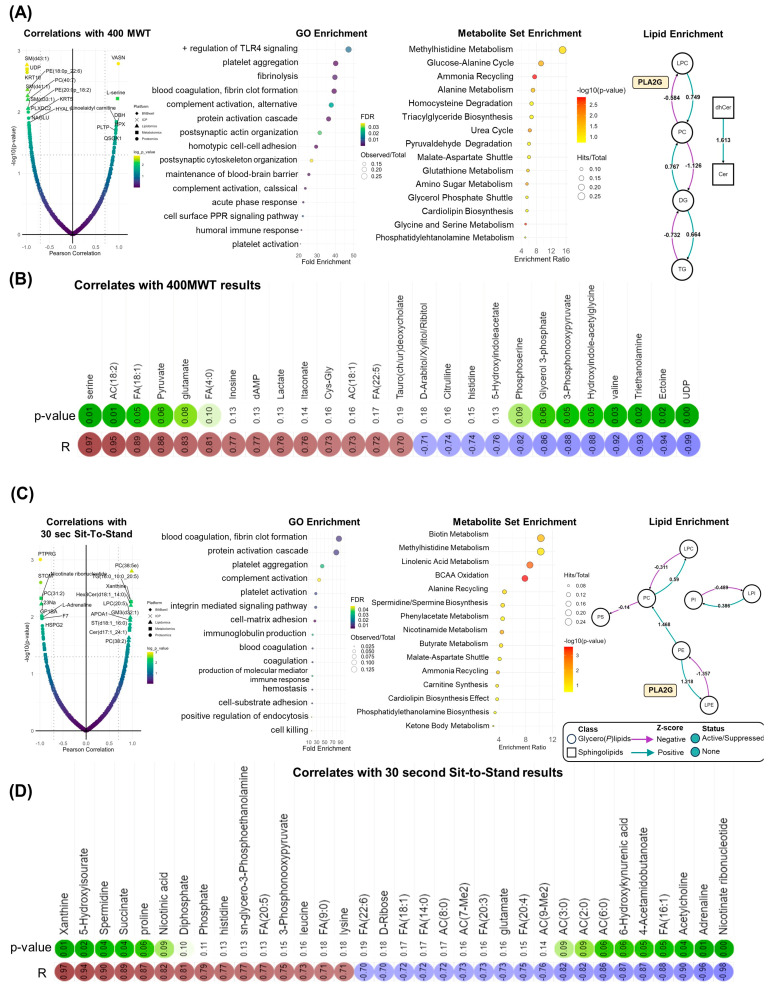
Correlation analysis with Performance Changes. (**A**) Total feature correlations are shown in the smile plot (left), gene ontology (GO) enrichment of proteomics data and metabolite set enrichment of metabolomics data are shown (middle) and lipid enrichment (right) are displayed for the 400 m walk test (400 MWT) results. (**B**) Pearson coefficients (R, in blue or red for negative or positive correlation, respectively) and corresponding *p*-values (significance highlighted in green) for metabolite correlates are shown. (**C**) The same systematic analysis is shown for 30 s sit-to-stand results and (**D**) corresponding correlations.

Comparable systematic patterns were observed when correlating molecular features with changes in physical function. Times for the 400 MWT were positively correlated with the TGFβ inhibitor vasorin (VASN), norepinephrine regulator dopamine beta hydroxylase (DBH), and heme scavenging protein hemopexin (HPX). In addition, positive associations between 400 MWT times and lactate, oleic acid (FA[18:1]), and linoleidyl carnitine (AC[18:2]) highlight improvements in aerobic capacity as the abundance of these compounds decreased with walk time (Figure 5A,B) [21]. Systematically, 400 MWT improvements correlated with proteins involved in immune function (e.g., toll-like receptor (TLR4) signaling) and hemostasis (e.g., platelet aggregation, coagulation). Nitrogen homeostasis centrality was observed in MSEA, highlighted by enrichment in amino acid, ammonia recycling, and urea cycle metabolism. Moreover, phospholipolysis mediated by phospholipase A2 (PLA2G) was revealed in lipidomics data (Figure 5A, right). Like 400 MWT, GO analysis of proteomic correlates with chair rise repetitions determined the enrichment of proteins involved in coagulation, hemostasis, and immune system activity including complement activity and immunoglobulin production (Figure 5C). In addition, 30 sec sit-to-stand results were inversely correlated with circulating acylcarnitines, which decreased in conjunction with increased repetitions. Higher repetitions were associated with increased levels of purine catabolites (xanthine, 5-hydroxyisourate), the polyamine spermidine, and TCA cycle intermediate succinate (Figure 5D).

## 4. Discussion

Exercise has been increasingly recognized as an effective therapy for cancer patients in general, and PDAC patients in particular, due to improved response to surgery and therapy, reduction in treatment side effects, and noted improvements in quality of life [33]. Moreover, while aerobic exercise reduces PDA tumor growth, prolongs survival, and sensitizes tumors to chemotherapy in animal models [34], more research is needed to understand the effects of exercise, and underlying mechanisms, in PDAC patients [35]. A recent clinical trial of resistance and aerobic exercise in PDAC patients (*n* = 64) receiving NAT demonstrated similar physical and functional improvements as this case series [9]. However, the authors are unaware of investigations into the mechanisms of these improvements, specifically using a multiomics approach. To date, the only study investigating exercise-associated mechanisms in this population demonstrated potential tumor vasculature remodeling [36]. Therefore, we describe here a proof of principal case study that leveraged state-of-the-art orthogonal mass spectrometry-based to analyze the plasma proteome, lipidome, metabolome, and metalome of PDAC patients before and after a personalized exercise program. Completion of this program resulted in improved physiological parameters such as lean mass and ASMI, as well as improved physical function as assessed by standardized 400 MWT and 30 s sit-to-stand tests [14]. Although limited in enrollment to three participants, this case study details a multiomics approach to provide quantitative information on 1053 molecules, which helped characterize molecular associations with physical improvements after exercise training. The validity of this approach is supported by protein and lipid correlates with fat mass. For instance, albumin has been shown to inversely correlate with fat percentage and body mass index in humans [37], an observation, which was reproduced here.

Among several observed pathways, systematic biological analyses suggested the impact that exercise training has on inflammation. Improved ASMI measurements were associated with an increase in immunoglobulin G (IgG), indicating that exercise in these patients might stimulate the adaptive immune system and potentially lead to better recognition and targeting of tumor cells. This adaptation would be particularly promising as PDAC tumors are largely considered immunologically “cold” tumors [38]. In addition, we observed a decrease in direct markers of inflammatory status including acute phase proteins C-reactive protein (CRP) and Serum Amyloid A (SAA1 and SAA2). This response indicates a reduction in systemic inflammation after the supervised exercise program, which is favorable as chronic inflammation can contribute to cancer progression [39]. On the other hand, positive correlations were observed between ASMI and alpha-2-Macroglobulin (A2M) and Alpha-2-HS-Glycoprotein (AHSG), which are protease inhibitors involved in regulating the coagulation cascade. The upregulation of these proteins might reflect a shift towards maintaining proteostasis, a crucial aspect of cellular homeostasis that, when disturbed, can contribute to cancer progression [40]. While immune cell phenotyping was not available for this study, future work should consider associations between multiomic signatures and immune cell populations. Indeed exercise-mediated impact on leukocyte populations is well established. Recent work has begun to demonstrate impacts in breast cancer patients as well, who exhibited exercise intensity-dependent leukocyte mobilization [41].

We also observed correlations between several small molecules, lipids, and functional performance metrics including the 400 MWT and 30 s sit-to-stand test. In the two patients who completed the exercise program, we observed an inverse correlation between medium-chain acylcarnitine levels and improvements in functional performance, indicating improvement in mitochondrial capacity after exercise training. Free fatty acids are mobilized for energy generation within the mitochondria via conjugation with L-carnitine to form acylcarnitines. Accumulation of these molecules in plasma, especially medium-chain acylcarnitines, are markers of fatigue in elite cyclists [21] and mitochondrial dysfunction in patients suffering from post-acute sequelae of COVID-19 (PASC) [42] or diabetes [43].

This case study serves as proof-of-concept for successfully performing the presented analyses in this under-investigated patient population with the associated exercise intervention. Associations were observed with clinical measures associated with survival and surgical outcomes, specifically physical fitness (400 MWT) and body composition (ASMI) [5,6]. Measurement of these immunological and mitochondrial pathways upon initiation of a preoperative exercise intervention could guide biological personalization of the exercise prescription by emphasizing resistance training to influence body composition or endurance training to influence mitochondrial function, as indicated. Tracking of these outcomes throughout the intervention could also provide real-time information on the effectiveness of the exercise prescription, with possible indications for prescription adaptation.

The primary limitation of this proof-of-concept study was low patient recruitment. Due to the limited number of participants in this study, systems analyses were performed on all five samples (including before and after samples for Participants 1 and 2, and only before for Participant 3). Moreover, the inclusion of all samples from participants in the data analysis highlighted that Participant 3 had higher inflammatory protein levels at the outset, including albumin, SAA1/2/4, and CRP. As a case study with only three subjects, it Is not possible to make broad conclusions regarding inflammatory status. However, these data do highlight the potential in monitoring patient status and forecasting exercise or treatment response, as it was discovered prior to surgery that Participant 3’s cancer had metastasized, and she was transitioned to palliative care. To increase enrollment, future investigations can be expanded through the advent of high-quality supervised telehealth exercise oncology programs [44,45], which were developed to overcome geographic accessibility issues in this population. The integrated utilization of remote dried blood sampling for mass spectrometry-based workflows [46,47,48], which are amenable to monitoring molecular responses to exercise [21,22], will enable the continued investigation of the findings presented in this study. The combination of these platforms improves access to supervised and omics-guided exercise training for cancer patients, thereby expanding both the data-driven resolution of this therapeutic intervention and diversifying the population of patients who may benefit from targeted exercise therapy.

## Figures and Tables

**Table 1:** Body Composition.

	Participant 1	Participant 2	Participant 3
Age	70 years	74 years	70 years
Sex	Male	Male	Female
BMI	20.8	24.8	16.3
Length of exercise intervention (weeks)	17	21	19
Exercise sessions (N)	28	55	46
NAT Description	Four 2-week cycles of FOLFIRINOX followed by 5 treatments with SBRT	Three 4-week cycles of gemcitabine/ABRAXANE followed by 5 treatments with SBRT	Four 2-week cycles of FOLFIRINOX followed by 5 treatments with SBRT

Abbreviation: BMI, body mass index. NAT (neo-adjuvant treatment).

**Table 2:** Physical Fitness.

	Participant 1			Participant 2			Participant 3	
	Baseline	Pre-Surgery	Post-Surgery	Baseline	Pre-Surgery	Post-Surgery	Baseline	Pre-Surgery
400 MWT (s)	211	188 (+11)	195 (+8)	213	205 (+4)	213 (0)	214	190 (+11)
30 s Sit-to-Stand Test	13	20 (+54)	43.5 (+8)	9	13 (+44)	10 (+11)	22	22 (0)
Total Mass (kg)	63.5	71.8 (+13)	64.7 (+2)	78.8	76.5 (−3)	69.2 (−12)	45.2	47.3 (+4)
Lean Mass (kg)	49.8	57.0 (+15)	51.3 (+3)	57.9	58.4 (+1)	54.1 (−6)	37.2	38.9 (+4)
Fat Mass (kg)	11.2	12.2 (+9)	10.8 (−4)	18.1	15.3 (−15)	12.5 (−31)	6.2	6.6 (+6)
Appendicular Lean Mass (kg)	21.7	25.6 (+18)	22.7 (+5)	23.9	25.6 (+7)	22.7 (−5)	15.1	15.5 (+3)
ASMI (kg/m^2^)	6.96 *	8.22 (+18)	7.28 (+6)	7.36	7.86 (+7)	6.98 * (−5)	5.3 *	5.45 (+3)

Abbreviation: 400 MWT, 400-m Walk Test. ASMI, Appendicular Skeletal Muscle Index. Percent changes indicated in parentheses. * Meets criteria for pancreatic cancer cachexia (<7.26 kg/m^2^ in males and <5.45 kg/m^2^ in females).

## Data Availability

The data presented in this study are available in the Supplementary Materials.

## Supplementary Materials

The following supporting information can be downloaded at: https://www.mdpi.com/article/10.3390/pathophysiology31010013/s1.

## Abbreviations

A2M	Alpha-2-Macroglobulin
AHSG	Alpha-2-HS-Glycoprotein
AC	Acylcarnitine
BMI	Body Mass Index
CD14	Cluster of Differentiation 14
CRP	C-Reactive Protein
DBH	Dopamine Beta Hydroxylase
DXA	Dual-Energy X-Ray Absorptiometry
GO	Gene Ontology
HCA	Hierarchical Clustering Analysis
HPX	Hemopexin
HRNR	Hornerin
ICP-MS	Inductively Coupled Plasma Mass Spectrometry
IGHV3-20	Immunoglobulin Heavy Variable 3-20
IGLV5-39	Immunoglobulin Lambda Variable 5-39
KRT2	Keratin 2
KRT33B	Keratin 33B
KRT6A	Keratin 6A
LC-MS	Liquid Chromatography-Mass Spectrometry
MBOAT7	Membrane Bound O-Acyltransferase Domain Containing 7
MSEA	Metabolite Set Enrichment Analysis
MWT	Meter Walk Test
NAT	Neoadjuvant Treatment
NOS	Nitric Oxide Synthase
PASC	Post-Acute Sequalae of COVID19
PC	Phosphocholine
PCA	Principal Component Analysis
PE	Phosphoethanolamine
PI	Phosphatidylinositol
PLA2G	Phospholipase A2 Group
PLS-DA	Partial Least Squares-Discriminant Analysis
PLXDC2	Plexin Domain Containing 2
QCs	Quality Controls
RPE	Rating of Perceived Exertion
SAA1	Serum Amyloid A1
SAA2	Serum Amyloid A2
SDS	Sodium Dodecyl Sulfate
SM	Sphingomyelin
SMPDB	Small Molecule Pathway Database
TEAB	Triethylammonium Bicarbonate
TLR4	Toll Like Receptor 4
UHPLC-MS	Ultra-High Performance Liquid Chromatography-Mass Spectrometry
VASN	Vasorin
